# Technology-Assisted Cognitive Motor Dual-Task Rehabilitation in Chronic Age-Related Conditions: Systematic Review

**DOI:** 10.2196/44484

**Published:** 2023-05-22

**Authors:** Cosimo Tuena, Francesca Borghesi, Francesca Bruni, Silvia Cavedoni, Sara Maestri, Giuseppe Riva, Mauro Tettamanti, Rosa Liperoti, Lorena Rossi, Maurizio Ferrarin, Marco Stramba-Badiale

**Affiliations:** 1 Applied Technology for Neuro-Psychology Lab Istituto Auxologico Italiano Istituto di Ricovero e Cura a Carattere Scientifico Milan Italy; 2 Department of Psychology University of Turin Turin Italy; 3 Faculty of Psychology Università eCampus Novedrate Italy; 4 Humane Technology Lab Università Cattolica del Sacro Cuore Milan Italy; 5 Laboratory of Geriatric Epidemiology Istituto di Ricerche Farmacologiche Mario Negri Istituto di Ricovero e Cura a Carattere Scientifico Milan Italy; 6 Fondazione Policlinico Universitario Agostino Gemelli Istituto di Ricovero e Cura a Carattere Scientifico Rome Italy; 7 Scientific Direction IRCCS INRCA Ancona Italy; 8 Fondazione Don Carlo Gnocchi Istituto di Ricovero e Cura a Carattere Scientifico Milan Italy; 9 Department of Geriatrics and Cardiovascular Medicine Istituto Auxologico Italiano Istituto di Ricovero e Cura a Carattere Scientifico Milan Italy

**Keywords:** dementia, Parkinson disease, falls, virtual reality, dual-task, aging, rehabilitation

## Abstract

**Background:**

Cognitive-motor dual-task (CMDT) is defined as the parallel processing of motor (eg, gait) and cognitive (eg, executive functions) activities and is an essential ability in daily life. Older adults living with frailty, chronic conditions (eg, neurodegenerative diseases), or multimorbidity pay high costs during CMDT. This can have serious consequences on the health and safety of older adults with chronic age-related conditions. However, CMDT rehabilitation can provide useful and effective therapies for these patients, particularly if delivered through technological devices.

**Objective:**

This review aims to describe the current technological applications, CMDT rehabilitative procedures, target populations, condition assessment, and efficacy and effectiveness of technology-assisted CMDT rehabilitation in chronic age-related conditions.

**Methods:**

We performed this systematic review, following PRISMA (Preferred Reporting Items for Systematic Reviews and Meta-Analyses) guidelines, on 3 databases (Web of Science, Embase, and PubMed). Original articles that were published in English; involved older adults (>65 years) with ≥1 chronic condition and/or frailty; and tested, with a clinical trial, a technology-assisted CMDT rehabilitation against a control condition were included. Risk of bias (Cochrane tool) and the RITES (Rating of Included Trials on the Efficacy-Effectiveness Spectrum) tool were used to evaluate the included studies.

**Results:**

A total of 1097 papers were screened, and 8 (0.73%) studies met the predefined inclusion criteria for this review. The target conditions for technology-assisted CMDT rehabilitation included Parkinson disease and dementia. However, little information regarding multimorbidity, chronicity, or frailty status is available. The primary outcomes included falls, balance, gait parameters, dual-task performance, and executive functions and attention. CMDT technology mainly consists of a motion-tracking system combined with virtual reality. CMDT rehabilitation involves different types of tasks (eg, obstacle negotiation and CMDT exercises). Compared with control conditions, CMDT training was found to be pleasant, safe, and effective particularly for dual-task performances, falls, gait, and cognition, and the effects were maintained at midterm follow-up.

**Conclusions:**

Despite further research being mandatory, technology-assisted CMDT rehabilitation is a promising method to enhance motor-cognitive functions in older adults with chronic conditions.

## Introduction

Carrying out cognitive and motor tasks simultaneously is essential for most daily activities. This ability allows people to, for instance, pay attention to the environment and avoid obstacles while walking [1,2]. The independent parallel performance of 2 tasks that can be measured separately and have distinct goals is defined as a dual-tasking situation [3]. In particular, a cognitive-motor dual-task (CMDT) is the simultaneous processing of a motor (eg, gait, gait initiation, balance, or physical exercise) and cognitive (eg, attention, decision-making, or working memory) activity [3,4]. Such an interplay between cognition and motor ability has been observed between some cognitive functions, such as attention and executive functions, and motor activities, such as gait, balance, and motor control [4-6]. The neural underpinnings of the CMDT are mainly located in the prefrontal regions of the brain [7,8]. Indeed, these regions are critical to support functions such as executive functions, attention, gait, and balance during dual-task activity [9]. However, when the demands of carrying out 2 tasks at once exceed cognitive motor skills, the performance on one or both tasks could be affected [4]. This finding supports the main theories on dual-task: the bottleneck and the limited attentional capacity sharing theories [9]. The latter posits that owing to a limited capacity of parallel processing, the performance of each task is reduced and at least one function is impaired. The bottleneck theory states that when 2 tasks recruit the same neural networks, one or both functions decline or become delayed.

Consequently, research has demonstrated that, compared with young adults, older adults pay a higher price for completing dual-task demands owing to deficits in motor and cognitive functions [8,10,11]. This is especially true for frail or multimorbid older adults [12-14]. This reduced ability can have serious consequences (eg, falls and injuries) for older adults [2]. Parallel to this, older adults aged ≥65 years with and without frailty or chronic diseases (eg, multimorbidity and dementia) are at great risk for falls and cognitive decline [15-20].

Nevertheless, the outcome of the CMDT training can be helpful for either one or both task-related functions and these beneficial effects can be used to train cognitive-motor functions [4,21]. CMDT rehabilitation is a therapeutic method that promotes the functioning of cognitive and motor skills [22]. The use of CMDT training could be beneficial given its effects on prefrontal cognitive and motor functions, which are affected by both normal and pathological aging [8,9,23,24].

A double-blind randomized controlled trial (RCT) by Silsupadol et al [25] showed that both dual- and single-task training were effective in improving balance during the single-task assessment in older adults aged ≥65 years with impaired balance; however, the dual-task intervention was superior to single-task training during dual-task assessment. A recent meta-analysis [22] showed that CMDT training is capable of improving different motor functions, such as gait and balance, compared with control conditions (eg, conventional therapy) in patients with chronic stroke. The meta-analytic work by Li et al [26] showed that dual-task is better than single-task conditions in enhancing motor and balance deficits in older adults with Parkinson disease (PD). A systematic review [27] has demonstrated that physical exercise combined with a cognitive task, compared with single (physical or cognitive) training, is more effective in enhancing prefrontal cognitive functions in older adults. A review by Gallou-Guyot et al [28] showed that CMDT exergames in healthy older adults were more effective in improving physical and especially cognitive functions than single-task or control conditions.

Despite being promising, the evidence in favor of CMDT rehabilitation can be improved by conducting more trials in older adults with and without chronic age-related conditions and by refining methodological (eg, RCT and type and structure of intervention) and technical aspects (eg, technological innovation) [22,27,29]. Concerning the latter point, CMDT can be carried out either with or without a technological system: dual-task can be performed, for instance, by walking in place and counting backward without the aid of any technological device to monitor the performance or to create interactive scenarios [3].

However, the use of innovative technological interfaces allows the patient to simultaneously perform motor and cognitive activities during CMDT. They enable one to track the participant’s performance, provide immediate feedback to the patient, and improve engagement [28,30,31]. A growing number of pilot and feasibility studies on CMDT training for age-related chronic conditions showed that motion-tracking systems can be successfully used to reproduce in immersive virtual reality (VR) or computer screen the movements performed by the patient while being involved in a cognitive task during the interaction with the immersive VR or computer screen [30,32-37]. In particular, immersive VR systems (eg, head-mounted displays and cave automated virtual environment) allow for the creation of compelling cognitive tasks similar to reality while interacting with the body and being stimulated in a multisensorial manner in the virtual world [38,39]. Other intriguing technological solutions, such as robots or home-based solutions, can aid the rehabilitation of cognitive-motor performance in aging by sustaining a motor function (eg, dual-task robotic-assisted gait) or long-term care (eg, dual-task telerehabilitation) [40-44].

Given the optimistic results of such pilot studies, further clinical trials are required to test the effects of CMDT rehabilitation with technological solutions (*technology-assisted CMDT rehabilitation*) in aging, both in clinical experimental and real-world settings. In particular, the management of chronic diseases and frailty in aging should be among the primary objectives of health care professionals and researchers [45].

To our knowledge, no systematic review has focused on technology-assisted CMDT rehabilitation in older adults with chronic conditions and frailty. Therefore, this paper aims to summarize the target populations, technological solutions, CMDT training characteristics, and efficacy or effectiveness of CMDT rehabilitation in age-related chronic conditions and to provide useful information for future research.

## Methods

### Literature Search

This systematic review was conducted (first search February 5, 2022, and updated on September 28, 2022) according to the PRISMA (Preferred Reporting Items for Systematic Reviews and Meta-Analyses) guidelines [46]. This review was registered at PROSPERO (registration number: CRD42022329783).

The keyword selection was carried out by following the population, intervention, comparison, outcome (PICO) guidelines [47], as follows: “frailty” OR “multimorbidity” OR “comorbidity” OR “chronic” AND “aging” OR “elder*” OR “old” (population keywords) AND “dual task” OR “dual-task” AND “robot” OR “information and communication technology” OR “assistive technologies” OR “social network” OR “smart homes” OR “ambient assisted living” OR “tele*” OR “medication optimization” OR “technology-based” OR “instrumented” OR “digital” OR “e-health” OR “machine learning” OR “artificial intelligence” OR “computer” OR “smartphone” OR “iPhone” OR “tablet” OR “touch screen” OR “iPad” OR “projectors” OR “CAVE” OR “visor” OR “head-mounted display” OR “oculus rift” OR “simulator” OR “virtual” OR “augmented reality” OR “accelerometer” OR “sensor*” OR “gyroscope” OR “magnetometers” OR “platform” OR “pressure insole” OR “pressure mat” OR “Kinect” OR “motion capture” OR “tracking” OR “exergame*” OR “treadmill” OR “software” OR “app*” OR “phone” (intervention keywords) AND “rehabilitation” OR “treatment” OR “rehab*” OR “management” OR “therapy” OR “training” OR “intervention” (outcome keywords).

We did not use the comparison term to gather all possible trials on technology-assisted CMDT rehabilitation. Thus, the PICO question was “Is technology assisted dual-task rehabilitation effective on subjects with age-related chronic conditions?” The combination of the cited keywords was performed in PubMed, Web of Science, and Embase and the research strategies were narrowed based on the titles and abstracts of the records. All the keywords except the technology keywords were searched in the databases for title, abstract, and keywords; the technology keywords were searched in the full text to include the highest number of papers that used technologies. After removing duplicates, 4 blinded researchers, in pairs (F Borghesi and F Bruni; SC and SM), used a web systematic review tool [48] to select records following the inclusion and exclusion criteria. The first selection was based on titles and abstracts, in which papers were considered as “included,” “excluded,” or “unsure.” The second selection considered full-text papers and reviewed those included in the first phase. The authors whose full-text papers were unavailable were contacted. In both phases, conflicts were resolved by consensus of the researchers and a third author (CT) was consulted for the remaining discrepancies.

### Selection Criteria

**Figure 1:**
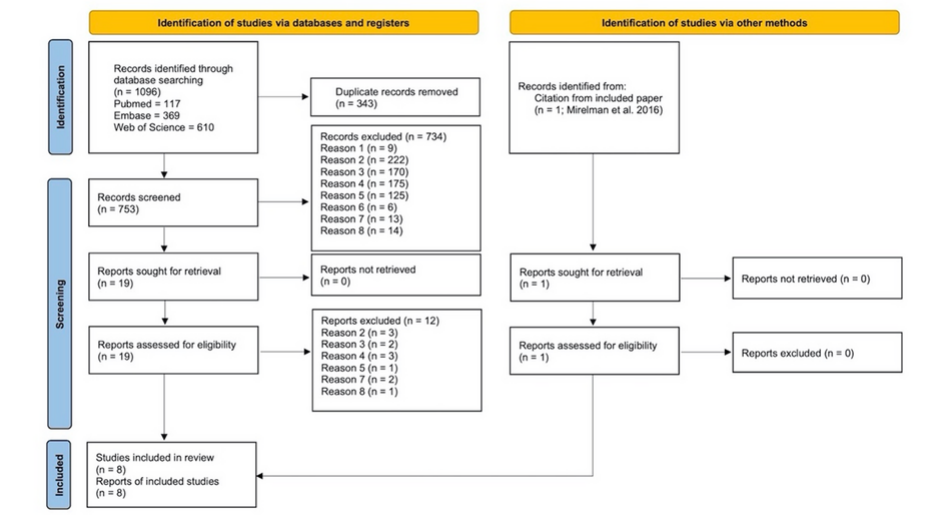
PRISMA (Preferred Reporting Items for Systematic Reviews and Meta-Analyses) flowchart of the included studies.

We adopted the following hierarchy of eligibility criteria for title and abstract and full-text screening (Figure 1 provides details on the number of excluded papers):

1. Articles in English
2. Original articles (no narrative or systematic reviews, meta-analyses, case reports, abstracts, conference proceedings, letters, or editorials)
3. Participants aged a mean of ≥65 years for the experimental and control conditions
4. Presence of ≥1 chronic conditions and/or frailty. A condition is classified as chronic if it is permanent, is caused by nonreversible pathological alterations, or requires rehabilitation or a long period of care [49]. Frailty was operationalized if assessed with any of the tools or criteria included in the systematic review by de Vries et al [50].
5. Experimental, quasiexperimental, and observational designs (no diagnostic, usability, or feasibility study assessment)
6. Presence of a control condition
7. CMDT training clearly explained or cited
8. Focused on a clear technology-assisted CMDT rehabilitation (see the keywords), that is, the technology is not just used to assess the dual-task ability or one of the functions; conversely, it is an interface used by the patient to perform the intervention

### Data Extraction and Synthesis

In each author pair (F Borghesi and F Bruni and SC and SM), one of the authors collected data for every study and then checked for accuracy and completeness. The following variables were extracted: clinical condition; multimorbidity, chronicity, or frailty condition assessment; primary outcome variables; CMDT technology; research methodology; dual-task and control condition description; number of treatment sessions; frequency of sessions; duration of each session; duration of the treatment; and efficacy. The results of the included studies are presented in Table 1.

**Table 1:** Summary of the included studies.

Study	Sample	Multimorbidity, chronicity, and frailty assessment	Primary outcomes	CMDT^a^ technology	Type of intervention	Session details	Main results
Mirelman et al [51], 2011	20 PD^b^	Disease duration	Usual gait Gait during DT^c^ Gait during endurance testing Obstacle negotiation TMT-AB^d^ UPDRS^e^ motor scores, FSST^f^, PDQ-39^g^	Semi-immersive VR^h^ motion system (V-TIME^i^ project)	Open-label trial CMDT group: walking on a treadmill while negotiating virtual obstacles presented on a screen in front of the treadmill. TAU^j^ group: historical active control group (physical treadmill training)	6 weeks: 18 sessions, 3 times a week for 45 minutes follow-up: 4 weeks	Similar results were obtained for the CMDT and TAU groups for DT gait parameters (eg, gait speed, stride length). However, gains were greater for the experimental group. Gait speed during usual walking, endurance, gait speed while obstacles negotiation increased after CMDT, and they were maintained over follow-up. DT gait speed and DT gait variability improved after training and at follow-up. Major improvements were found also in TMT-AB, UPDRS motor scores, FSST, and PDQ-39.
Mirelman et al [20], 2016	282 patients: 130 PD, 43 MCI^k^, 109 with IF^l^	PD duration, number of prescribed medications, history of falls	Incident rate of falls in the 6 months after the end of the training	Semi-immersive VR motion system (V-TIME project)	RCT^m^ CMDT group: walking on a treadmill while negotiating virtual obstacles presented on a screen in front of the treadmill. TAU group: walking on a treadmill	6 weeks: 18 sessions, 3 times a week for 45 minutes Follow-up: 6 months	CMDT outperforms TAU condition in reducing fall incident rate, especially in PD. Obstacle clearance was improved in the CMDT compared with TAU. Endurance, obstacle clearance, mobility, and quality of life were maintained at follow-up in the CMDT group. Gait speed during usual and obstacle negotiation gait, cognitive functions, and physical performance improved in both groups.
Delbroek et al [52], 2017	20 MCI	Not reported	MoCA^n^ POMA^o^ and iTUG^p^ iTUG during DT	VR exergame (BioRescue)	RCT CMDT group: 9 exercises, including obstacle negotiation, weight-bearing transfer exercises, and cognitive operation with weight-bearing exercises. TAU group: low-intensity strength and flexibility exercises for the upper body while seated.	6 weeks: 12 sessions 2 times a week. The duration gradually increased from 18 minutes (week 1) to 30 minutes (week 5) No follow-up	iTUG parameters improved for the CMDT compared with TAU. No changes in the POMA, iTUG, and MoCA scores in both groups were observed.
Wiloth et al [13], 2017	99 cognitive impairment (probable dementia)	Number of medications and diagnoses, history of falls, living situation	Physiomat performance (trained and untrained)	VR exergame (Physiomat)	RCT CMDT group: by shifting weight while holding onto the handles of the Physiomat, participants had to complete 2 cognitive tasks FTBT^q^: moving a yellow ball on the screen as fast as possible from the center of the screen to the target items PTMTs^r^: connect numbers provided as fast as possible on 5 different levels. TAU group: nonspecific, low-intensity training on strength and flexibility for the upper body while seated.	10 weeks: 20 sessions, 2 times a week for 90 minutes Follow-up: 3 months	Trained and untrained FTBT and PTMTs parameters (eg, accuracy, time) improved after CMDT compared with TAU group. Effects were maintained at follow-up for trained tasks.
Maidan et al [53], 2018	64 PD	Disease duration	Prefrontal HbO2^s^ with f-NIRS^t^ and gait were assessed during 3 walking tasks: usual walking, walking while serially subtracting 3 seconds from a given 3-digit number (DT), and walking while negotiating obstacles Incident rate of falls in the 6 months after the end of the training Neuropsychological test battery	Semi-immersive VR motion system (V-TIME project)	RCT CMDT group: walking on a treadmill while negotiating virtual obstacles presented on a screen in front of the treadmill. TAU group: walking on a treadmill.	6 weeks: 18 sessions, 3 times a week for 45 minutes Follow-up: 6 months	CMDT reduced prefrontal activation during usual walking and complex walking conditions compared with TAU. EF^u^ and falls incident rate at 6 months improved in the CMDT group. Both interventions improved gait parameters (eg, speed, stride length).
Werner et al [54], 2018	99 cognitive impairment (probable dementia)	Number of medications and diagnoses, history of falls, living situation	Physiomat performance (FTBT, PTMTs)	VR exergame (Physiomat)	Secondary analyses of an RCT CMDT group: see Wiloth et al [13], 2017. ERs^v^: participants with an individual decrease in the duration after TS7^w^ that exceeded the RCI^x^ either for the most complex Physiomat task completed at T1^y^ or for at least 50% of the Physiomat tasks completed at T1. NERs^z^: all other participants. TAU group: low-intensity strength and flexibility exercises for the upper body while seated.	10 weeks: 20 sessions, 2 times a week for 90 minutes No follow-up	From T1 to TS7: substantial improvements in the FTBT and PTMT level 1, 2, and 3-5. From TS7 to TS14: substantial improvements in the PTMT level 2 and 4. From TS14 to T2: substantial improvements in the PTMT level 1 and 4. From TS7 to T2: substantial improvements in the FTBT and PTMT level 1 and 3-5. ERs had lower visuospatial ability, DT performance, and Physiomat compared with NERs. The predictors of an early CMDT response are visuospatial ability, DT performance, and Physiomat
Pelosin et al [55], 2018	96 PD	Disease duration, history of falls	Gait under dual-task condition EF Incident rate of falls in the 6 months after the end of the training	Semi-immersive VR motion system (V-TIME project)	RCT 6 versus 12 weeks of CMDT training aided with technology CMDT group: walking on a treadmill while avoiding virtual obstacles projected on the screen.	6 weeks: 18 sessions, 3 times a week for 45 minutes Follow-up: 1 and 6 months 12 weeks: 32 sessions, 3 times a week for 45 minutes Follow-up: 1 and 6 months	Both interventions improved, and maintained over follow-up, gait parameters during DT, obstacle negotiation, or usual gait. EF improved at posttest and 1-week follow-up for the 12-week CMDT. Incident rate of falls improved in both groups, but results were stronger for the 12-week interventions. Fear of falling improved and was maintained in the 12-week program
Spanò et al [34], 2022	26 CVD^aa^ with history of falls	No detailed information reported	Balance and gait (also DT) Fear of falling Physical performance and gait speed	Semi-immersive virtual room with a motion-tracking system	RCT pilot study CMDT group: simultaneous administration of motor and cognitive tasks, with sensory carpets and with walkable LED floor. TAU group: individual underwent combined motor and cognitive training.	DT: 5 weeks: 15 sessions, 3 times a week for 40 minutes No follow-up TAU: 5 weeks: 15 sessions, 3 times a week for 60 minutes total	CMDT improved gait and balance (POMA) and fear of falling but not gait endurance. TAU did not show any improvements. Gait under DT was improved in the CMDT group only. No other effects were found.

^a^CMDT: cognitive-motor dual-task.

^b^PD: Parkinson disease.

^c^DT: dual-task.

^d^TMT-AB: Trail Making Test A/B.

^e^UPDRS: Unified Parkinson’s Disease Rating Scale.

^f^FSST: four square step test.

^g^PDQ-39: Parkinson’s Disease Questionnaire.

^h^VR: virtual reality.

^i^V-TIME: Virtual Reality-Treadmill Combined Intervention for Enhancing Mobility and Reducing Falls in the Elderly.

^j^TAU: treatment as usual.

^k^MCI: mild cognitive impairment.

^l^IF: idiopathic falls.

^m^RCT: randomized control trial.

^n^MoCA: Montreal Cognitive Assessment.

^o^POMA: performance-oriented mobility assessment.

^p^iTUG: instrumented Timed Up and Go.

^q^FTBT: follow the ball task.

^r^PTMT: trail making task.

^s^HbO_2_: oxyhemoglobin.

^t^f-NIRS: functional near-infrared spectroscopy.

^u^EF: executive function.

^v^ER: early responders.

^w^TS: training session.

^x^RCI: reliable change index.

^y^T1: baseline.

^z^NER: nonearly responder.

^aa^CVD: cerebrovascular disease.

### Quality Assessment

Interventional randomized clinical trials were assessed using the Cochrane Collaboration’s risk of bias tool [56]. This tool allows for the assessment of the following sources of bias as “high risk,” “low risk,” “unclear risk,” or “risk not applicable”: (1) selection bias referring to randomization procedures and allocation concealment, (2) performance bias regarding blinding of participants and research staff, (3) detection bias concerning blinding of assessors and data handlers and analysts, (4) attrition bias referring to dropouts and missing data, and (5) reporting bias concerning systematic errors in reporting study outcomes.

One nonrandomized study was assessed using the risk of bias in nonrandomized studies of interventions [57]. This tool evaluates seven domains through which bias can occur, depending on the stage of intervention: (1) preintervention—at this stage, the risk of bias assessment is mainly distinct from the assessment of RCTs; the domains evaluated were the bias owing to confounding and bias in the selection of participants; (2) intervention—this phase is also distinct from the assessment of RCTs and addresses the bias in the classification of interventions; (3) postintervention—at this stage, the risk of bias substantially overlaps with the assessment of RCTs. The scale evaluates 4 domains: bias owing to deviations from intended interventions, bias owing to missing data, bias in the measurement of outcomes, and bias in the selection of the reported results. The evaluation of each domain is guided by questions that facilitate judgments, leading to one of the following responses: “yes,” “probably yes,” “probably no,” “no,” and “no information.” These responses lead to domain-level judgments about the risk of bias, which then allow an overall risk of bias evaluation: “low risk,” “moderate risk,” “serious risk,” and “critical risk” of bias.

Four blinded researchers, in 2 pairs (F Borghesi and F Bruni and SC and SM), evaluated independently the quality of the studies. Conflicts were resolved by consensus of authors in each pair or by the involvement of a third author (CT) in case of discrepancies. Table 2 provides the risk of bias assessment results for each item. Regarding the nonrandomized study, the study by Mirelman et al [51] showed low risk for most of the scale items (bias owing to confounding, selection of the participants, classification of the interventions, deviations from the intended interventions, missing data, and selection of the reported results) beside a high risk in the “bias in measurements of outcomes” item.

**Table 2:** Randomized controlled trial risk of bias assessment.

Study	Random sequence generation	Allocation concealment	Blinding of participants and personnel	Blinding of outcome assessment	Incomplete outcome data	Selective reporting
Mirelman et al [20], 2016	Low risk	Unsure	High risk	Low risk	Low risk	Low risk
Delbroek et al [52], 2017	Unsure	Unsure	Low risk	Low risk	Unsure	Low risk
Wiloth et al [13], 2017	Low risk	High risk	Low risk	Low risk	Low risk	Low risk
Maidan et al [53], 2018	Unsure	High risk	High risk	Low risk	Low risk	Low risk
Werner et al [54], 2018	Low risk	Unsure	Low risk	Unsure	Low risk	Low risk
Pelosin et al [55], 2018	Low risk	High risk	High risk	Low risk	Low risk	Low risk
Spanò et al [34], 2022	High risk	High risk	High risk	High risk	Low risk	Low risk

### The Efficacy-Effectiveness Spectrum of the Trials

We evaluated the efficacy-effectiveness of the included trials according to the RITES (Rating of Included Trials on the Efficacy-Effectiveness Spectrum) tool [58]. It allows the characterization of randomized trials included in a systematic review on an efficacy-effectiveness continuum to understand whether a trial is potentially useful to inform clinical decision-making in usual care. The RITES tool contains 4 domains (participants’ characteristics, trial setting, flexibility of intervention, and clinical relevance of experimental and comparison intervention), each rated on a 5-point scale from a strong emphasis on efficacy to a strong emphasis on effectiveness.

**Figure 2:**
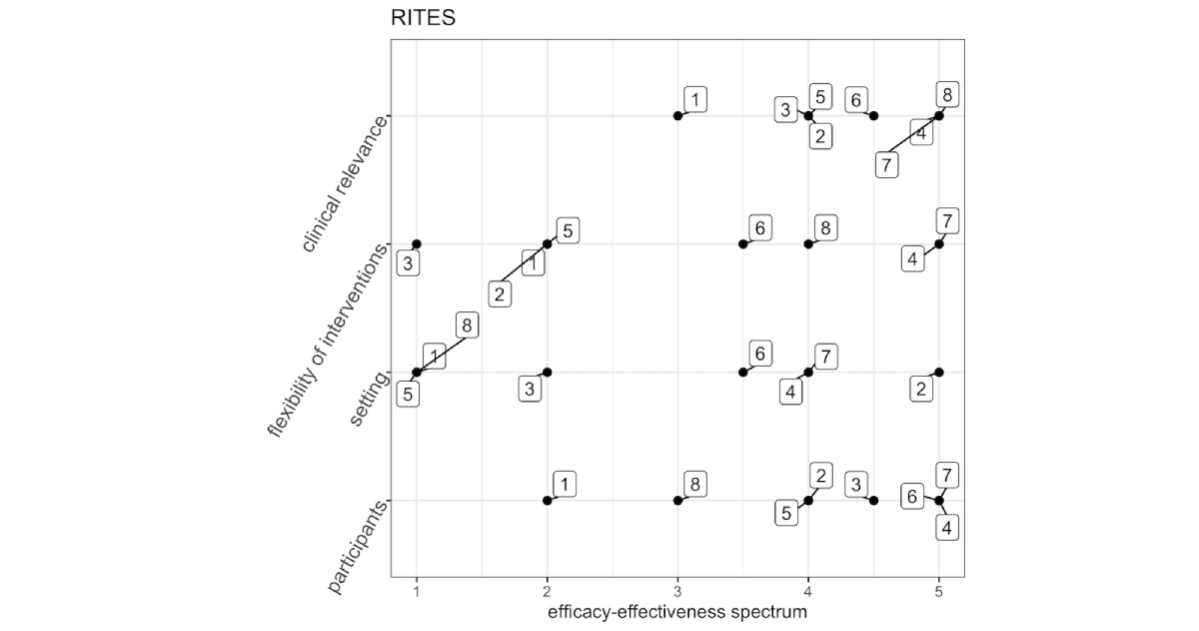
Visual presentation of the efficacy-effectiveness of trials; boxes represent each study. 1: Mirelman et al [57], 2011; 2: Delbroek et al [53], 2017; 3: Wiloth et al [13], 2017; 4: Maidan et al [54], 2018; 5: Werner et al [55], 2018; 6: Spanò et al [34], 2022; 7: Pelosin et al [56], 2018; 8: Mirelman et al [20], 2016. RITES: Rating of Included Trials on the Efficacy-Effectiveness Spectrum.

Each researcher in each blinded pair expressed their opinion related to the domains of the RITES, collecting 4 different judgment ratings from 1 to 5. Each pair then collected the mean values of their judgment for every domain. Figure 2 displays the average of the ratings by the 2 judges for each domain for each trial.

## Results

### Clinical Population and Disease Assessment

Three studies assessed the efficacy of technology-assisted CMDT rehabilitation in PD [51,53,55]. The Unified Parkinson’s Disease Rating Scale [51,53,59] and the UK Parkinson’s Disease Society Brain Bank Criteria [55,60] were used to diagnose PD. One study [34] evaluated the impact of technology-assisted CMDT rehabilitation in patients with chronic cerebrovascular disease who are at risk of falling. The diagnosis was made using medical and fall history and by using the Tinetti performance-oriented mobility assessment [61]. Three studies recruited older adults with different degrees of cognitive and motor impairment [13,52,54]. One study [52] involved institutionalized older adults who were able to walk 10 m with walking aids and with mild cognitive impairment as assessed with the Montreal Cognitive Assessment [62]. The RCT by Wiloth et al [13] and its secondary analysis [54] included individuals who were able to walk 10 m without walking aids and with cognitive impairment (ie, probable dementia), as assessed by the mini–mental status examination [63] and criteria of the consortium to establish a registry for Alzheimer disease [64]. The RCT by Mirelman et al [20] recruited older adults with a history of falls (idiopathic falls) and individuals with PD or mild cognitive impairment. PD was diagnosed according to the UK Parkinson’s Disease Society Brain Bank Criteria [60], whereas mild cognitive impairment was diagnosed using the clinical dementia rating scale (threshold score of 0.5) [65].

Information regarding the multimorbidity, chronicity, and frailty status of the sample was obtained from the included studies. Disease duration was included as a baseline measure in all PD studies and the absence of severe concurrent medical conditions was applied as an exclusion criterion [20,51,53,55]. Two studies balanced the groups of frail and multimorbid patients with cognitive impairment, considering the severity of depressive symptoms, history of falls, and number of concurrent diagnoses and medications [13,54]. One RCT [20] controlled the experimental and control groups for, among other measures, the number of medications, fall history, and global cognition. However, frailty was not assessed with any of the tools or criteria included in the systematic review by de Vries et al [50]. No detailed information regarding frailty status, morbidity, or chronicity was found in 2 studies [34,52].

### Primary Outcomes of the Trials

Most of the studies aimed at evaluating pre-post improvement in both cognitive and motor aspects. Falls, balance, gait parameters, dual-task performance, and executive functions and attention were of particular interest.

Falls were evaluated as a reduction in frequency at 6 months (incident rate) after the end of the intervention in 3 studies [20,53,55]. In the study by Spanò et al [34], the falls efficacy scale-international [66] was used.

Balance and gait were assessed using the Tinetti scale [61] and the instrumented Timed Up and Go test [67] by Delbroek et al [52]. Spanò et al [34] assessed balance and gait with the Tinetti scale [61] and the 6-minute walking test [68].

One study [34] asked participants to perform subtractions during the primary outcome measures assessment. The instrumented Timed Up and Go test was combined with a picture-name matching task to evaluate motor-cognitive dual-task ability [52]. Physiomat motor-cognitive dual-task performance at the *follow the ball task* (FTBT) and *trail making tasks* (PTMTs) were used in 2 studies [13,54]. Werner et al [54] performed an additional dual-task assessment (walking while performing a working memory task). Intriguingly, Maidan et al [53] assessed the prefrontal cortex HbO_2_ with functional near-infrared spectroscopy while walking under 3 conditions, including preferred speed, dual-task (walking plus subtractions), and obstacle negotiation. Pelosin et al [55] used the spatiotemporal parameters of dual-task (walking plus verbal fluency) as primary gait outcome and the preferred speed and obstacle negotiation as secondary measures. Mirelman et al [51] assessed gait under different conditions, including usual speed, dual-task (walking plus subtraction), obstacle negotiation, and gait endurance with the 6-minute walking test [68].

Cognition (with a focus on executive functions) was assessed with a complete computerized battery (Mindstreams; NeuroTrax Corp) in 2 studies [53,55]. The Trail Making Test (part A and B) was used by Mirelman et al [51], whereas 1 study [52] used the Montreal Cognitive Assessment [62] to assess executive functions and global cognition.

### Dual-Task Technology

All the studies used an interactive technological system (motion-tracking system with VR) for CMDT training.

Two studies [13,54] proposed an exergame-based balance training system using Physiomat. It is a device consisting of a 3D movable plate with integrated sensors for displacement measurement. It is connected to a computer and a screen. Patients can grab the rails on each side to ensure their stability during training and assessment. To solve a Physiomat game task shown on a computer screen, the user must control and move the cursor by bending, tilting, and rotating movements of their feet while standing on a balance platform movable in the sagittal, frontal, and transversal plane.

In 4 studies [20,51,53,55], a semi-immersive VR system was developed (V-TIME [Virtual Reality-Treadmill Combined Intervention for Enhancing Mobility and Reducing Falls in the Elderly] project [69]). Participants walked on the treadmill with a safety harness while viewing the virtual environment on a large screen. LEDs were attached to the lateral side of each participant’s usual shoes to track foot movement.

One study [34] used a semi-immersive dual-task virtual room. It included 3 sensory carpets (2 m; medium density; smooth, sandy, and cobbled), a video projector, 5 screens, a walkable LED floor (4.5 m × 1.5 m), and an audio or video controller console.

Delbroek et al [52] used BioRescue, a semi-immersive VR-based program for CMDT rehabilitation. It has a platform (610×580×10 mm^3^) equipped with 1.600 pressure sensors that measure vertical pressure fluctuations in both feet. During the training, BioRescue provided the participants with real-time feedback on a screen about the movement of the center of pressure.

### Type and Structure of the Interventions

In this study, we only included studies with a control condition. Treatment as usual (TAU) consisted of treadmill physical training in 3 studies [20,53,55]. One study [51] used a historical (treadmill physical training) active control group from a previous work [70]. Furthermore, 3 studies [13,52,54] used low-intensity strength and flexibility exercises for the upper body without technology as a TAU condition. However, Werner et al [54] focused on the early and late technology-assisted CMDT rehabilitation responders comparison. One study [34] used as a TAU group a combined but separate cognitive (computerized) and motor (without technology) training.

Three studies used a technology-assisted CMDT obstacle negotiation training [51,53,55]. Two studies applied 2 technology-assisted CMDT exercises with Physiomat [13,54]—the FTBT (moving a yellow ball on the screen as fast as possible from the center of the screen to the target items; weight-bearing transfer exercises) and the PTMTs (connecting numbers provided as fast as possible on 5 different levels; cognitive operation with weight-bearing exercises). One study [52] used the dual-task exercises included in the BioRescue program (eg, obstacle negotiation, weight-bearing transfer exercises, and cognitive operation with weight-bearing exercises). The research by Spanò et al [34] used different cognitive-motor exercises with the sensory carpet (eg, following the traffic lights, the environmental scenarios inclusive of the congruent and incongruent sounds, the association of sounds and images to remember, walking while looking for numbers, and making calculations) and LED wall with projectors (eg, go/no-go, walking Stroop, and walking Trail Making Test). The exercises stimulate different cognitive domains and motor abilities.

The duration of the interventions ranged from 6 to 12 weeks, the total number of sessions ranged from 12 to 32, the number of sessions per week from 2 to 3, and the session duration ranged from 18 to 90 minutes [13,20,34,51-55]. Follow-ups ranged from 1 to 6 months [13,20,51,53-55]. Two studies [34,52] did not include a follow-up.

### Results of the CMDT Trials

In the study by Spanò et al [34], the technology-assisted CMDT rehabilitation improved the Tinetti total, balance, and gait scores and the fear of falling but not the 6-minute walking test. No effects were observed in the TAU group. Dual-task performance improved only in the technology-assisted CMDT rehabilitation group for the Tinetti total and gait scores; no other effects were found.

In the study by Delbroek et al [52], the technology-assisted CMDT rehabilitation improved the instrumented timed up-and-go total time and the turn-to-sit transition. However, the step time before the turn worsened in the experimental group. No other considerable differences were observed.

Wiloth et al [13] found that Physiomat CMDT training improved both trained and untrained FTBT and PTMTs (all complexity levels). At the 3-month follow-up, all the trained tasks were sustained. The secondary analysis [54] showed a substantial training session improvement in the FTBT and PTMTs levels. In particular, early responders were found to have lower global cognition, FTBT, visuospatial ability, processing speed, and dual-task performance at baseline compared with nonearly responders. Physiomat, visuospatial ability, and dual-task score were predictors of early training response. This suggests that individuals with cognitive impairment and lower dual-task ability respond faster to technology-assisted CMDT training.

The work by Mirelman et al [51] showed that compared with the historical active control group, gait speed and stride length were similar to the technology-assisted CMDT rehabilitation; however, the effects were larger in the latter group. Usual gait, dual-task gait, gait endurance, and obstacle negotiation scores were improved at the posttest and were maintained at follow-up. The Trail Making Test improved at posttest but could not be compared with a control condition, as was absent in the active control group study.

The RCT by Mirelman et al [20] found that the falls incident ratio was reduced in the VR CMDT intervention compared with the TAU group (also adjusting from global cognition) in the sample regardless of the subsample (older people with a history of falls, PD, and mild cognitive impairment). Individuals with PD benefited the most from the VR training (also when adjusting for disease severity), whereas healthy older adults with a history of falls and mild cognitive impairment showed comparable results from the 2 interventions. Among the secondary outcomes, gait parameters under the obstacle negotiation condition were better for the technology-assisted CMDT rehabilitation group than in the TAU group. However, speed under the normal walking condition, physical performance, and cognition improved in both treatments. At the 6-month follow-up, obstacle clearance, mobility, endurance, and quality of life were maintained for the technology-assisted CMDT condition.

The work by Pelosin et al [55] showed only a main effect of time (also at a 6-month follow-up) on gait parameters under different conditions (6- vs 12-week technology-assisted CMDT training). Executive functions improved in the 12-week program and were maintained at 1-month follow-up but not at 6-month follow-up. Attention and processing speed improved with both interventions. Falls were reduced in the 6 months after both training sessions and particularly for the 12-week program.

The prefrontal activity was reduced in the technology-assisted CMDT training compared with the TAU condition, particularly for the left cortex [53]. However, the right prefrontal cortex activity during the complex walking conditions was reduced for the dual-task and obstacle negotiation. Gait parameters, falls, and executive functions improved for both training programs. The reduced activity found in this study suggests that technology-assisted CMDT rehabilitation reduces compensatory neurophysiological hyperactivation because of PD.

Table 3 reports substantial results of the primary outcomes in favor of CMDT training compared with TAU conditions of the trials. Overall, there is promising evidence that CMDT rehabilitation could be an innovative method to improve dual-task and motor abilities in age-related conditions, at least right after the intervention. Further evidence on mid- and long-term maintenance of the improvements is needed. However, although the effect size of some studies is convincing, several studies did not report this parameter; this hampers a clear and rigorous understanding of CMDT rehabilitation efficacy or effectiveness.

Adherence was >80% in some studies [13,20,34,54,55] but was not reported in the remaining studies. One study [52] rated emotions during the technology-assisted CMDT or TAU rehabilitation. They found that during the BioRescue training, alertness and pleasure were observed and the patients reported it as a useful intervention. One study [13] showed that the number of adverse events was the same for Physiomat and TAU. The secondary analysis of this trial [54] showed that 9% (4/45) of participants dropped out before the seventh session.

**Table 3:** Primary outcome results in favor of CMDT^a^ rehabilitation compared with TAU^b^.

Study and primary outcomes			Sample		Pretest, mean (SD)		Posttest, mean (SD)		Follow-up, mean (SD)		*P* value		Effect size
**Spanò et al [34], 2022**													
	POMA^c^ total score	26 CVD^d^		CMDT: 18.8 (2.6); TAU: 18.8 (6.7)		CMDT: 23 (2.6); TAU: 20.1 (6.4)		Not applicable		.01		Not reported	
	POMA balance score	26 CVD		CMDT: 10.4 (2.0); TAU: 11 (4.6)		CMDT: 12.8 (2.0); TAU: 12.0 (4.1)		Not applicable		.03		Not reported	
	POMA gait score	26 CVD		CMDT: 8.4 (1.7); TAU: 7.8 (2.6)		CMDT: 10.2 (1.2); TAU: 8.1 (2.9)		Not applicable		.01		Not reported	
**Delbroek** **et al** **[52],** **2017**													
	iTUG^e^ total durations	20 MCI^f^		CMDT: 17.2 (9.0); TAU: 22.1 (13.8)		CMDT: 15.8 (9.2); TAU: 20.1 (9.8)		Not applicable		.02		Not reported	
	iTUG turn-to-sit durations	20 MCI		CMDT: 5.3 (2.5); TAU: 6.4 (3.8)		CMDT: 4.6 (2.0); TAU: 6.4 (3.3)		Not applicable		.02		Not reported	
	iTUG step time before turns	20 MCI		CMDT: 0.7 (0.2); TAU: 0.6 (0.1)		CMDT: 0.5 (0.2); TAU: 0.6 (0.1)		Not applicable		.02		Not reported	
**Wiloth** **et al** **[13],** **2017**													
	Trained FTBT^g^ durations	99 cognitive impairment		CMDT: 30.9 (17.5); TAU: 28.9 (15.7)		CMDT: 19.3 (4.6); TAU: 23.4 (5.5)		6 months later, CMDT: 20.8 (6.5); TAU: 22.3 (5.4)		T1^h^-T2^i^: *P*<.001; T1-T3^j^: *P*<.05		T1-T2: η^2^_p_=0.25; T1-T3: η^2^_p_=0.05	
	Trained FTBT accuracy (digits/ms)	99 cognitive impairment		CMDT: 4450.4 (2859.8); TAU: 4164.3 (3922.4)		CMDT: 3169.7 (557.2); TAU: 3776.3 (1286.9)		6 months later, CMDT: 3166.8 (658.4); TAU: 3498.8 (824.4)		T1-T2: *P*>.001; T1-T3: *P*<.038		T1-T2: η^2^_p_=0.14; T1-T3: η^2^_p_=0.06	
	Trained PTMT^k^ score (points)	99 cognitive impairment		CMDT: 3.4 (1.4); TAU: 3.2 (1.5)		CMDT: 4.7 (0.9); TAU: 3.6 (1.4)		6 months later, CMDT: 4.5 (1.0); TAU: 4.0 (1.1)		T1-T2: *P*>.001; T1-T3: not significant		T1-T2: η^2^_p_=0.21	
	Untrained PTMT score (points)	99 cognitive impairment		CMDT: 3.1 (1.4); TAU 3 (1.5)		CMDT: 4.5 (1.0); TAU 3.5 (1.4)		6 months later, CMDT: 4.3 (1.2); TAU: 3.9 (1.2)		T1-T2: *P*<.001; T1-T3: not significant		T1-T2: η^2^_p_=0.18	
**Mirelman et al [51], 2011**													
	Dual-task gait	20 PD^l^		CMDT: 13.9 (14.8); TAU: historical active control group		CMDT: 6.9 (8.4); TAU: historical active control group		Not applicable		.05		Not reported	
**Mirelman et al [20], 2016**													
	Incident rate falls	282 PD, MCI, and patients with IF^m^		CMDT: 11.92 (9.47-15.01); TAU: 10.71 (8.51-13.47)^n^		Not applicable		6 months, CMDT: 6 (4.36-8.25); TAU 8.27 (5.55-12.31)^n^		.03		Incident rate ratio=0.58	
**Maidan et al [53], 2018**													
	Prefrontal activation during walking	64 PD		Not reported		Not reported		Not applicable		.01		Not reported	
	Lateralization activation (left vs right) dual-task gait	64 PD		Not reported		Not reported		Not applicable		.04		Not reported	
	Lateralization activation (left vs right) obstacle negotiation	64 PD		Not reported		Not reported		Not applicable		.02		Not reported	

^a^CMDT: cognitive-motor dual-task.

^b^TAU: treatment as usual.

^c^POMA: performance-oriented mobility assessment.

^d^CVD: cerebrovascular disease.

^e^iTUG: instrumented Timed Up and Go.

^f^MCI: mild cognitive impairment.

^g^FTBT: follow the ball task.

^h^T1: pretest.

^i^T2: posttest.

^j^T3: follow-up.

^k^PTMT: trail making task.

^l^PD: Parkinson disease.

^m^IF: idiopathic falls.

^n^Values report incident rate.

### Efficacy-Effectiveness Spectrum

The results of the RITES tool showed interesting results concerning the items assessed using this tool.

#### Participants’ Characteristics

High scores on the RITES tool indicate that the participants are representative of the population who would receive the experimental intervention if it was part of usual care. Low scores on the RITES tool indicate that the participants have completed a careful selection and present homogeneous characteristics, different from those observed in a clinical population or usual care. Moreover, 4 studies [13,34,53,55] had a strong emphasis on effectiveness and 2 studies [52,54] had a rather strong emphasis on effectiveness (4-point Likert scale). One study [20] had a balanced efficacy-effectiveness score. One study [51] had a rather strong emphasis on efficacy (2-point Likert scale). None of the studies had a strong emphasis on efficacy (1-point Likert scale).

#### Trial Setting

High RITES scores indicate that the trial setting is similar to usual care and might include multiple subsettings that replicate usual care. Low RITES scores indicate a setting that replicates experimental conditions as in laboratories or academic centers. Only 1 study [52] had a strong emphasis on effectiveness (5-point Likert scale). Two studies [53,55] had a rather strong emphasis on effectiveness (4-point Likert scale). One study [34] had a balanced emphasis on both efficacy and effectiveness (3.5-point Likert scale). Only 1 study [13] had a rather strong emphasis on efficacy (2-point Likert scale). Furthermore, 3 studies [20,51,54] had a strong emphasis on efficacy (1-point Likert scale).

#### Flexibility of Interventions

Low RITES scores indicate that experimental and comparison intervention delivery is less flexible than the usual care. High RITES scores indicate that flexibility in the experimental and comparison interventions was identical to that in usual care. Only 1 study [13] had a strong emphasis on efficacy (1-point Likert scale). Another study [34] had a balanced emphasis on both efficacy and effectiveness (3.5-point Likert scale). One study [20] had a rather strong emphasis on effectiveness. Two studies [53,55] had a strong emphasis on effectiveness (5-point Likert scale).

#### Clinical Relevance of Experimental and Comparison Interventions

High RITES scores indicate that both the experimental and comparison interventions have the potential to be “best practice.” The duration of the interventions was similar to the minimum length of treatment in the usual care. Instead, low RITES scores indicate that one or both experimental and comparison interventions are not clinically relevant or that the best current treatment or study duration is shorter than the minimum length of treatment in usual care. Three studies [20,53,55] had a strong emphasis on effectiveness. Four studies [13,34,52,54] had a rather strong emphasis on effectiveness. Only Mirelman et al [51] had balanced emphasis on both efficacy and effectiveness. Most of the studies are more toward clinical effectiveness.

Overall, the above picture suggests that most of the studies included patients whose characteristics resembled those seen in the usual care. This also reflects on the flexibility of the interventions provided, which can be adjusted according to the patient’s needs and clinical characteristics. The studies appear equally distributed in terms of the study setting in which training could be carried out. More importantly, almost all the studies indicate that both the experimental and control conditions have the potential for being “best practice.” Therefore, the studies included show that the research in the field of CMDT rehabilitation for chronic age-related conditions is progressively more oriented toward usual care conditions and a greater external validity of the interventions.

## Discussion

In this systematic review, we sought to describe the current applications of technology-assisted CMDT rehabilitation in older adults with chronic conditions and/or frailty.

We observed that (1) the target conditions were PD and cognitive impairment; however, only some studies provided information regarding multimorbidity, chronicity, or frailty; (2) the primary outcomes of interest were falls, balance, gait parameters, dual-task performance under different conditions (eg, usual speed, dual-task, obstacle, and Physiomat tasks), and cognition (eg, executive functions and attention); (3) CMDT technology mainly consisted of a motion-tracking system with semi-immersive VR or computer screen; (4) TAU conditions consisted of active control conditions (eg, low-intensity physical exercise with treadmill and nontechnological interventions), whereas technology-assisted CMDT rehabilitation consisted of different types of tasks (eg, obstacle negotiation, Physiomat tasks, and CMDT exercises); and (5) technology-assisted CMDT training was found to be pleasant, safe, and effective particularly for dual-task measures, falls, gait, and cognition, and the effects were maintained at midterm follow-ups.

Regarding the efficacy-effectiveness spectrum, we found that participants’ characteristics and clinical relevance of the included studies were mostly representative of usual care or real-world practice. Conversely, the setting (eg, specialized vs as usual care) and type of intervention (eg, strict vs flexible protocol) of the papers are rather heterogeneous and vary across the trials. Concerning the risk of bias assessment, the highest source of bias was randomization, allocation, and blinding of the participants.

The risk of cognitive and motor decline is high in healthy, frail, and multimorbid older adults (>65 years) and people living with dementia [15-20]. In addition, normal and pathological aging are characterized by a decline in dual-task ability [8,10,20]. Crucially, 1 study [13] showed that older people with deficits in cognition and dual-task respond faster to the technology-assisted CMDT therapy. Therefore, it is crucial to design and deliver technology-assisted CMDT rehabilitation programs for older adults living with chronic conditions and frailty.

Recent systematic reviews in aging and chronic diseases [22,26-28] have showed that CMDT interventions are beneficial for cognitive and motor function. The efficacy and engagement of CMDT rehabilitation could be improved by adopting interactive technological systems that enable one to monitor, aid, and empower cognitive-motor functions in aging [28,30,31]. In particular, VR equipped with motion sensors allows for the design of rehabilitative scenarios that involve the participants in cognitive and motor activity in a multisensory way and close-to-real-world conditions [38,39]. Indeed, we found that in older adults living with PD and cognitive impairment, technology-assisted CMDT rehabilitation is more effective in improving motor (eg, falls, balance, and gait), cognitive (eg, executive functions), and CMDT performance compared with TAU (ie, non–dual-task technological rehabilitation intervention) at short- and midterm assessment. In addition, it is safe and feasible and rated as pleasant by the patients. Intriguingly, one study [53] found that technology-assisted CMDT training is capable of promoting brain plasticity in the prefrontal cortex, which is crucial to sustain executive functions, motor functions, and dual-task activity [9]. Regarding the dose response of technology-assisted CMDT training, 1 study [55] showed that both 6- and 12-week interventions are effective but 12-week interventions are preferred.

In addition, the RITES evaluation showed that technology-assisted CMDT rehabilitation could be a feasible method to be implemented in usual care scenarios; however, before being included among usual care treatments, more studies (especially RCT) are required to test the efficacy and security and usability studies are required to improve technology acceptance by the patients.

On the basis of the evidence found, we provide the following recommendations for future geriatric research and practice:

1. Current applications are designed for heterogeneous clinical conditions (PD, mild cognitive impairment, and cerebrovascular disease) with some clinical overlap; however, to achieve more rigorous and consistent findings, more CMDT trials are needed.
2. Parameters of morbidity, chronicity, and frailty should be considered more in depth during the assessment (eg, baseline assessment), selection (eg, inclusion and exclusion criteria), and analyses (eg, covariates); indeed, such variables could interfere with CMDT efficacy or effectiveness, or frail and multimorbid individuals might be a preferred population for cognitive-motor interventions, given the prognostic impact of these geriatric conditions.
3. Primary outcomes of a CMDT trial should include motor, cognitive, and dual-task performances; the lack of one of these variables in the protocol might underestimate the efficacy of the treatment investigated (pre-post neurophysiological outcomes could also provide interesting information).
4. Future studies should consider to adopt innovative CMDT technologies (eg, immersive VR, robot-assisted rehabilitation, and home-based rehabilitation) to exploit their potential in geriatric conditions.
5. Further studies are required to understand the optimal dose-response relationship for CMDT interventions.
6. RITES evaluation shows that future trials should focus on the development of rigorous RCT methodology, although pragmatic and observational trials could provide the real-world impact of CMDT training solutions in geriatric patients.
7. CMDT rehabilitation provides a multicomponent and multidomain approach to geriatric conditions with tasks related to real-life situations that can be integrated with innovative computational approaches such as artificial intelligence, which can analyze a large amount of data for diagnostic, prognostic, and treatment monitoring purposes [71,72].

This review had several limitations. First, a few studies matched the keywords and stringent selection criteria; indeed, only 8 papers matched our search. Two papers [54,55] did not directly compare the experimental condition with a TAU condition but compared it with a CMDT (technology assisted) control condition. In addition, we did not evaluate the effect size and pooled efficacy of the trials included as the primary outcome of this study was to map the literature and evaluate the possibility to carry out a meta-analysis. Consequently, no direct conclusion regarding efficacy could be drawn.

In conclusion, technology-assisted CMDT rehabilitation is a prospective, powerful method that can be used to improve motor, cognitive, and CMDT performance in age-related chronic conditions. Despite these promising results, further trials are mandatory to support the efficacy or effectiveness, safety, and engagement of technology-assisted CMDT rehabilitation.

## Abbreviations

CMDT: cognitive-motor dual-task
FTBT: follow the ball task
PD: Parkinson disease
PICO: population, intervention, comparison, outcome
PRISMA: Preferred Reporting Items for Systematic Reviews and Meta-Analyses
PTMT: trail making task
RCT: randomized controlled trial
RITES: Rating of Included Trials on the Efficacy-Effectiveness Spectrum
TAU: treatment as usual
VR: virtual reality
V-TIME: Virtual Reality-Treadmill Combined Intervention for Enhancing Mobility and Reducing Falls in the Elderly
