# Correction: Technology-Assisted Cognitive Motor Dual-Task Rehabilitation in Chronic Age-Related Conditions: Systematic Review

**DOI:** 10.2196/51591

**Published:** 2023-09-26

**Authors:** Cosimo Tuena, Francesca Borghesi, Francesca Bruni, Silvia Cavedoni, Sara Maestri, Giuseppe Riva, Mauro Tettamanti, Rosa Liperoti, Lorena Rossi, Maurizio Ferrarin, Marco Stramba-Badiale

**Affiliations:** 1 Applied Technology for Neuro-Psychology Lab Istituto Auxologico Italiano Istituto di Ricovero e Cura a Carattere Scientifico Milan Italy; 2 Department of Psychology University of Turin Turin Italy; 3 Faculty of Psychology Università eCampus Novedrate Italy; 4 Humane Technology Lab Università Cattolica del Sacro Cuore Milan Italy; 5 Laboratory of Geriatric Epidemiology Istituto di Ricerche Farmacologiche Mario Negri Istituto di Ricovero e Cura a Carattere Scientifico Milan Italy; 6 Fondazione Policlinico Universitario Agostino Gemelli Istituto di Ricovero e Cura a Carattere Scientifico Rome Italy; 7 Scientific Direction IRCCS INRCA Ancona Italy; 8 Fondazione Don Carlo Gnocchi Istituto di Ricovero e Cura a Carattere Scientifico Milan Italy; 9 Department of Geriatrics and Cardiovascular Medicine Istituto Auxologico Italiano Istituto di Ricovero e Cura a Carattere Scientifico Milan Italy

In “Technology-Assisted Cognitive Motor Dual-Task Rehabilitation in Chronic Age-Related Conditions: Systematic Review” (J Med Internet Res 2023;25:e44484) the authors noted one error:

Affiliation of author Lorena Rossi:

“Scientific Direction, Istituto Nazionale Riposo e Cura Per Anziani, Istituto di Ricovero e Cura a Carattere Scientifico, Ancona, Italy”

has been replaced by:

“Scientific Direction, IRCCS INRCA, Ancona, Italy”.

The correction will appear in the online version of the paper on the JMIR Publications website on September 26, 2023, together with the publication of this correction notice. Because this was made after submission to PubMed, PubMed Central, and other full-text repositories, the corrected article has also been resubmitted to those repositories.

